# Gate Tuning of Förster Resonance Energy Transfer in a Graphene - Quantum Dot FET Photo-Detector

**DOI:** 10.1038/srep28224

**Published:** 2016-06-20

**Authors:** Ruifeng Li, Lorenz Maximilian Schneider, Wolfram Heimbrodt, Huizhen Wu, Martin Koch, Arash Rahimi-Iman

**Affiliations:** 1 Department of Physics and the State Key Laboratory of Silicon Materials, Zhejiang University, Hangzhou, 310027, P.R. China; 2 Faculty of Physics and Materials Sciences Center, Philipps-Universität Marburg, 35032 Marburg, Germany

## Abstract

Graphene photo-detectors functionalized by colloidal quantum dots (cQDs) have been demonstrated to show effective photo-detection. Although the transfer of charge carriers or energy from the cQDs to graphene is not sufficiently understood, it is clear that the mechanism and efficiency of the transfer depends on the morphology of the interface between cQDs and graphene, which is determined by the shell of the cQDs in combination with its ligands. Here, we present a study of a graphene field-effect transistor (FET), which is functionalized by long-ligand CdSe/ZnS core/shell cQDs. Time-resolved photo-luminescence from the cQDs as a function of the applied gate voltage has been investigated in order to probe transfer dynamics in this system. Thereby, a clear modification of the photo-luminescence lifetime has been observed, indicating a change of the decay channels. Furthermore, we provide responsivities under a Förster-like energy transfer model as a function of the gate voltage in support of our findings. The model shows that by applying a back-gate voltage to the photo-detector, the absorption can be tuned with respect to the photo-luminescence of the cQDs. This leads to a tunable energy transfer rate across the interface of the photo-detector, which offers an opportunity to optimize the photo-detection.

In recent years, graphene has been widely used for the development of new electro-optical devices such as transparent electrodes^1^, photovoltaic modules^2^, optical modulators^3^ and plasmonic devices^4^,^5^,^6^. Graphene, with its remarkable electronic and optical properties, opens an important avenue on high performance photo-detection. A plethora of studies focus on graphene photo-detectors because of its broad spectral bandwidth and high electron mobility. However, the weak light absorption limits its application for visible-range photo-detection. Hence, a combination of graphene with an excellent light absorber is necessary for the fabrication of efficient photodetectors. Several approaches have been published including a graphene semiconductor heterojunction^7^,^8^,^9^,^10^, graphene p-n junction^11^, graphene-quantum dots hybrid detectors^12^,^13^,^14^,^15^,^16^,^17^, graphene-transition-metal dichalogenides-graphene heterostructures^18^,^19^ and many more. The colloidal quantum dots (cQDs) hybrid structure delivers a promising candidate among them, where cQDs provide wavelength tunablility as well as an efficient light-absorbing capability, which will benefit the photo-detection, if one combines the optical advantages of cQDs together with the remarkable electrical properties of graphene. Such graphene-cQDs hybrid detectors are based on a layer of cQDs on top of the graphene, which absorb optical power and transfer the energy to the graphene. Such detectors can exhibit a high external quantum efficiency of up to 25%^16^. In this scenario, high gain is caused by a change of the carrier density in the graphene due to transfer of charge carriers or energy from the photo-excited cQDs across the interface with graphene. Nevertheless, the mechanism of transfer, especially the manipulation of transfer rates via an applied gate voltage, is not yet understood.

In general, the transfer consists of two parts: the charge transfer/Dexter transfer^20^ and the near field interaction/Förster resonance energy transfer (FRET)^21^,^22^,^23^. The Dexter transfer originates from the overlap of molecular orbitals. In this case, the electron transfer is limited to short distances which are of the order of less than one nanometre. In contrast, the Förster transfer is based on a near-field Coulomb interaction between two media across an interface, in which the working distance can amount to several nanometres. Indeed, in a practical system, both transfer mechanisms may coexist and contribute to the observed effects to a certain amount, but it is understandable that for rather short or large distances, one type predominantly determines transfer processes. For instance, the transfer of energy from functionalized quantum dots to dye molecules has been under investigation for many years. For such molecular assemblies, it has been shown that a modelling with a Förster transfer like model is possible^24^,^25^,^26^. Most recently, the case of transfer via short ligands dominated by Dexter transfer was investigated by Spirito *et al*.^27^. Naturally, in the intermediate regime, both mechanism of transfer have to be considered. In our work on a graphene-cQDs system, we investigate the contrasting case of long ligands expected to be dominated by Förster transfer.

Herein, we first built up a graphene field-effect transistor (FET) via a simple laser ablation method, which avoids a chemical photo-lithography process. We measured the time-resolved photo-luminescence (TRPL) on a graphene FET functionalized with CdSe/ZnS QDs at different back-gate voltages. A Förster transfer model is elucidated from the back-gate dependence of the PL lifetime. The optimization of the back-gate voltage for an efficient energy transfer is discussed together with a consideration of the device’s responsivity.

## Results and Discussion

**Figure 1:**
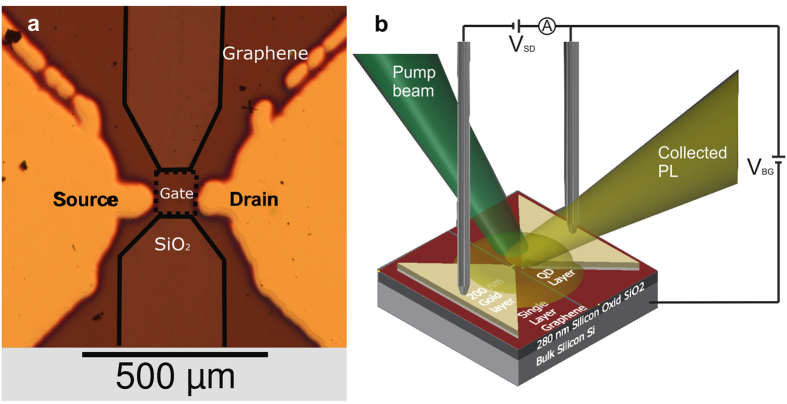
The graphene field-effect transistor structure. (**a**) Top view of the sample and the transistor geometry with labels projected onto a microscope image of one device. For clarity, the SiO_2_ region not covered by graphene is highlighted by a solid black line and the gate is highlighted by a dotted line. (**b**) Schematic three-dimensional representation of a fabricated graphene transistor comprising of the substrate layers, its contact pads and the illuminated graphene gate with a light-absorbing cQDs layer deposited on top of it.

The cQDs employed exhibit a PL peak at 580 nm and the first exciton absorbance peak at 560 nm. The characterization of absorbance as well as the photo-luminescence of these cQDs in solution are provided in the Supporting Information (cf. Fig. SI1a). Moreover, the radiative lifetime was analysed (cf. Fig. SI1b). From such experiments, two time constants were extracted using a bi-exponential fit of the normalized transient. Here, we yield a short lifetime of *τ*_1_ = 30 ± 1 ns and a slow decay with *τ*_2_ = 7.0 ± 0.5 μs. A graphene FET was fabricated using laser ablation and electrical contacts (Ti/Au:20 nm/200 nm) were deposited using metal evaporation in combination with a laser-cut shadow mask. Figure 1a presents a microscope image of such a processed graphene device. In a consecutive step, the cQDs were drop-casted on the patterned graphene area between the two metal contacts (see sample preparation in the method section). Thereafter, the FET has been contacted and time-resolved photo-luminescence measurements have been performed in dependency of the applied gate voltage (cf. Figs SI2, SI3 and the method section for further details). A schematic representation of the investigated cQDs-graphene system is shown in Fig. 1b.

**Figure 2:**
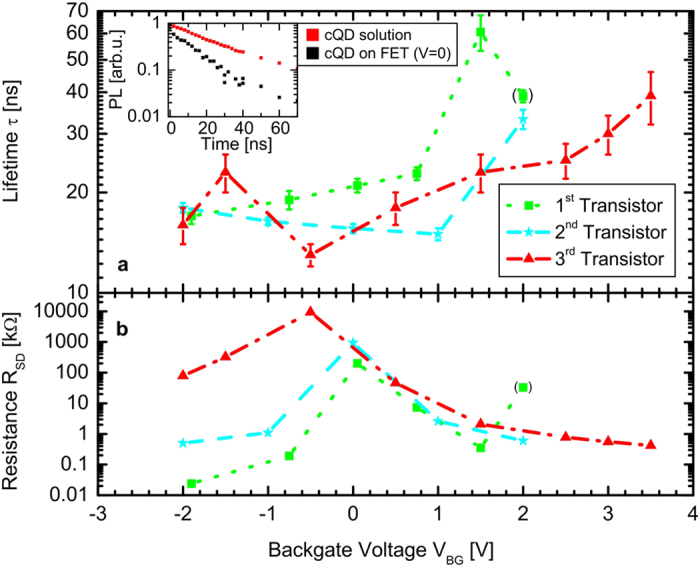
Gate-voltage dependent cQD photoluminescence lifetime and graphene resistance. Lifetimes of cQDs’ PL extracted from mono-exponential fits (cf. Fig. SI3) to the transients (**a**) and measured channel resistances (**b**) in dependence of the gating voltage, respectively, for three independent measurements on different transistors (red, green and blue symbols/curves, respectively). Error bars are displayed for the lifetimes according to the uncertainty parameter of the fit. In this study, the Dirac point of the transistors is slightly different in each case owing to a varying amount of cQDs deposited onto the gate region. The Dirac point is clearly observed for each transistor, and a slight asymmetry is obtained in the course of the recorded resistance. Furthermore, an increase of PL lifetime upon increased back-gate voltages is observed. Inset: Transients of cQD PL with (on FET at no bias) and without graphene, respectively.

To obtain insight into the transfer dynamics, three independent measurements with different but similar transistors were performed for the statistics, which demonstrate qualitative accordance. Indeed, those devices, which are based on the same design and are the result of the same fabrication technique, exhibited a similar structure as well as cQD coverage within fabrication tolerances (cf. Figs SI4 and SI5). The recorded transients were fitted with a mono-exponential decay (cf. Fig. SI3) neglecting the slow decay occurring at large delay times. We thereby found that the resulting lifetimes and the measured resistances in the graphene channel clearly depend on the gate voltage, as shown in Fig. 2a,b, respectively. The inset of Fig. 2 highlights a reduction of the short lifetime in transient PL data for cQDs on top of graphene at zero bias in contrast to pure cQDs. In each measured data set, for which individual TRPL measurements per gate-voltage setting were performed (cf. Fig. SI3), a drastic change of radiative lifetime is obvious. To be more specific, the difference between the shortest and the longest decay time amount on average to 266% relative to the fastest decay time. For any of the investigated cQD-covered transistors, one clearly obtains an increase in the lifetime for large positive applied gate voltages, while the corresponding resistance curve peaks at the Dirac-point of graphene.

**Figure 3:**
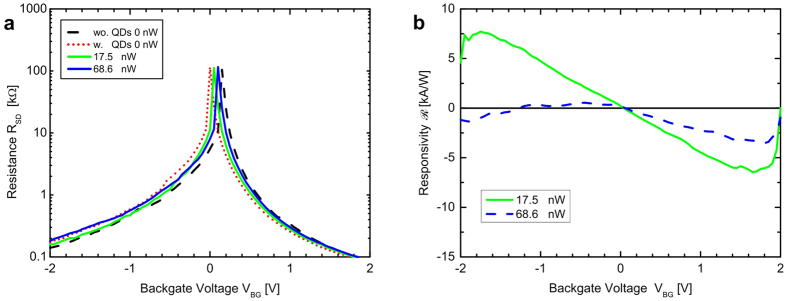
Responsivity of the graphene-based photo-detector structure. (**a**) Source-drain resistance response as a function of the back-gate voltage for the pure transistor (dark response only, dashed line) as well as for the cQD-functionalized transistor (dark response presented by dotted line) for different pump intensities (straight lines). The dark response of the functionalized Graphene with QDs is shifted relative to the pure graphene to negative voltages. For increasing optical power, the curve shifts back to higher voltages. The resulting responsivities as a function of the back-gate voltage are shown in (**b**). The point of zero responsivity is observed close to the Dirac point, where the curves in (**a**) cut the dark response curve. The point of highest responsivity is found at about ±1.7 V and an additional decrease of responsivity is observed towards ±2.0 V.

Furthermore, the shift of the Fermi level depending on the optical power was investigated for one of the transistors representatively. The resistance-response curve was therefore measured at different optical powers *P*. We can observe a shift of the response curve at functionalization as well as at different light intensities, as shown in Fig. 3a. With the same aim as presented in the work of Spirito *et al*.^27^, we calculated the responsivity as R = (*I*_*ph*_ − *I*_*dark*_)/(*P*), with *I*_*ph*_ representing the current at given illumination (*P*), and *I*_*dark*_ the FET current in the dark, respectively. Such responsivity curve is plotted in Fig. 3b. This chart shows that the performance of the device peaks at a back-gate voltage of ±1.7 V and decreases for an increased back-gate voltage. Furthermore, we observe zero responsivity at gate voltages close to the Dirac point owing to the high resistivity of graphene at corresponding bias. In comparison to the results presented by Spirito *et al*.^27^, we have observed the same behaviour, however on a smaller scale, since we have been investigating a system tailored to obtain and study the less efficient Förster transfer.

### Modelling

Since we have been investigating a system with long ligands (oleic acid), we expect the transfer to be dominated by a Förster-like energy transfer^23^. In the following, the main ideas of the Förster model shall be explained prior to theoretical modelling of the data using measurement-based parameters. This transfer model is based on the near-field interaction between two dipoles. If an excited carrier in the QD is close enough (below ~10 nm) to an unexcited carrier, which can absorb the excitation energy, it can pass on its energy through a non-radiative near-field interaction. The transfer rate *ω*_*FRET*_ is therefore proportional to the integral of the photo-luminescence of the quantum dots *PL*_*QD*_ (a measure of the number of excited cQDs) and the absorbance of graphene *α*_*Gr*_ (see equation (1)):


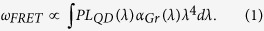


This means that the transfer rate peaks, when the high absorbance of graphene overlaps with the PL energy of cQDs, and decreases, when the absorbance of graphene mismatches the PL energy of QDs at a certain energy detuning. If the back-gate voltage *V*_*BG*_ is changed, the Fermi level *E*_*F*_ in the graphene is being tuned and the absorbance changes accordingly. In other words, by changing the back-gate voltage, one shifts the absorbance of graphene with respect to the fixed radiative transition energy, i.e. PL line, of the cQDs. The absorbance of graphene *α*(*ω*) consists of two parts, as equation (2) presents: the frequency (*ω*)-dependent intraband absorbance *α*_*intra*_(*ω*) and the interband absorbance *α*_*inter*_(*ω*).


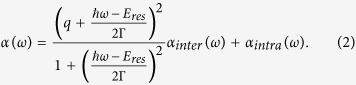


To include the effect of the exciton resonance, the interband absorbance has been multiplied by the fanofunction according to ref. 28 (see equation (2)) with Γ = 0.78, *q* = −1, *E*_*res*_ = 5.02*eV.* The interband absorbance can be modelled by equation (3)^28^ with *E*_*F*_ relative to the Dirac point in the frequency-dependent optical sheet conductivity *σ*(*ω*), with (*π*^2^*e*^2^)/(*ch*) comprising of natural constants (elementary charge *e*, speed of light *c* and Planck’s constant *h*, respectively) and *k*_*B*_*T* the thermal energy at room temperature:


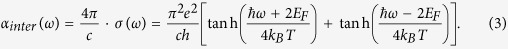


The intraband part can be modelled by a Drude model^28^. This has been calculated according to equation (4) with *τ*_*s*_ = 50 fs representing the momentum relaxation time at charge neutrality^28^:


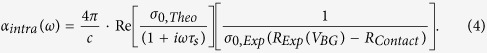


The measured conductance *σ*_0,*Exp*_ –conductivity at the Dirac point, represented by 1/*R*_*Exp*_(0*V*) –has been normalized to the theoretical value 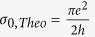 to neglect geometrical parameters. The magnitude of experimental conductivity times measured resistance (*R*_*Exp*_(*V*_*BG*_) − *R*_*Contact*_), which takes into account the estimated contact resistance of each device, is particularly used to estimate the uncertainty of the model values extracted for the absorbance.

**Figure 4:**
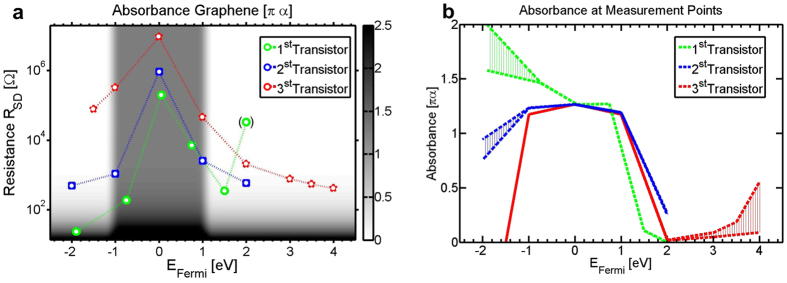
Absorbance of graphene. (**a**) Diagram of the mean theoretical absorbance of graphene from 530 nm to 640 nm as a function of the Fermi level, relative to the Dirac point, and the resistance according to eqations 1, 2, 3, 4, 5, 6. Furthermore, the chart indicates the measured points, where transients were acquired, from which corresponding absorbance values can be extracted. The absorbance is plotted in units of the theoretical absorbance of graphene corresponding to *πα* = 0.023. (**b**) Considering possible contact resistances for the estimation of uncertainties, the absorbance values are evaluated along those measured paths obtained in the overlap of data and model in (**a**). A plateau of high absorbance is found near the Dirac Point, which starts decreasing at about 1.1 eV. It reaches negligible absorbance at about 2 V. In the negative direction, the absorbance of two measurements start to decrease in the acquired data range, while one increases further.

The absorbance *α*(*ω*) given by equation (2) has been calculated in dependence of the gate voltage for wavelengths *λ* = 2*πc*/*ω* in the range of 530 nm to 640 nm (corresponding to the cQD emission line) and is plotted in Fig. 4a as a mean absorbance for this wavelength range. Thereby, a theoretical absorbance map is spanned as a function of the transistor’s source-drain resistance values *R*_*SD*_ paired with Fermi energies. In addition, the displayed chart in Fig. 4a indicates the measured points, where transients were acquired. Here, the x-axis represents the relative Fermi energy, which equals zero at the peak of the resistance curves for each transistor. The corresponding calculated absorbance values at these measured pairs of gate voltage, i.e. Fermi energy, and source-drain resistance *R*_*Exp*_(*V*_*BG*_) are shown in Fig. 4b. Here, the spread of lines represents the estimated uncertainty of the calculated values. Note that, in accordance to literature, absorbance of graphene is not provided in units of 1/cm, but as = *−*log(I/I_0_) in units of *πα* = 0.023. Here, the relative absorbance only considering interband transisions amounts to 1, while *α* of equation (2) exceeds unity due to its intraband component.

The measured photo-luminescence intensity can be described by an effective lifetime *τ*_*eff*_ consisting of two parts similar to Niebling *et al*.^24^ (see equation (5) and (6)). It consists of the pure lifetime *τ*_*QD*,*pure*_ of the cQDs (cf. Fig. SI1b) in solution and the transfer rate *ω*_*FRET*_ (see equation (1)), which was calculated as an integral over the aforementioned wavelength range of interest. In equation (6), *N*_*QD*_ is a factor that corresponds to the number of excited carriers in the cQDs.


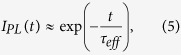



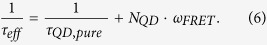


**Figure 5:**
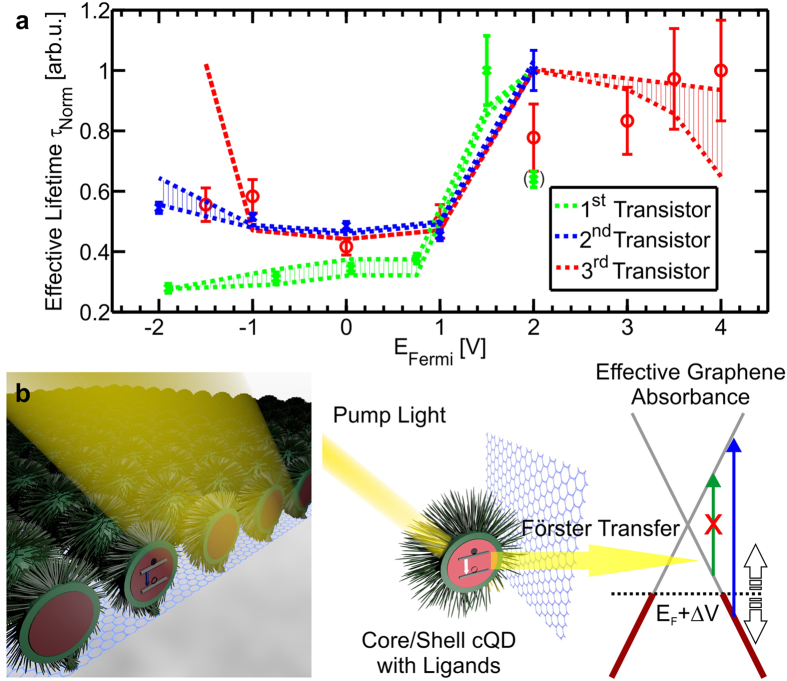
Gate tuning of Förster transfer at cQD-graphene interface. **(a**) Comparison of the normalized theoretical model for a Förster transfer based on measured parameters (lines) with the experimentally obtained data representing a measure of transfer rates (symbols). (**b**) Schematic picture of the effective Förster energy transfer. The photo excited carriers within the cQDs transfer their energy non-radiatively to the graphene, through the layer of ligands surrounding the excited core/shell structure, if the graphene can absorb it. The right part shows a sketch of the graphene band structure indicating the interband transitions that are feasible depending on the Fermi level.

From the calculated absorbance values, the effective radiative lifetime has been calculated according to equation (6) and plotted in Fig. 5 together with experimental data for all three devices. In order to compare experimental with modelled data, here, both results have been normalized to the maximum of each curve. Remarkably, the model describes the modification of the lifetime well, suggesting that the transfer is indeed dominated by a Förster transfer process. The model predicts that a plateau of high transfer rate is expected in a small range around the Dirac point (cf. Figs 4a and 5b).

To put this into context with the photo-detecting properties of our device, it is important to note that this region corresponds to a high resistance of graphene, implying that carriers are transferred to the graphene, but cannot be efficiently extracted from the gate region. This indeed explains the low responsivity obtained around the Dirac point and its surrounding maxima (cf. Fig. 3b).

Moreover, the model predicts that the transfer vanishes at voltages higher then ±2 V, for the considered cQD-graphene system. Comparing this outcome with the measured responsivity curve, we indeed observe a decay of responsivity at such high voltages. This model clearly predicts non vanishing transfer rates in the intermediate regime between ±2 V and the Dirac point, where the F*ET al*ready exhibits low source-drain resistances of the graphene layer, which explains that the maximum responsivities locate at about ±1.7 V in our case. The schematic picture of the cQD-graphene system representing the Förster-like energy transfer in that intermediate regime with variable gate voltages is shown in Fig. 5b.

## Conclusion

To summarize, in our work, we studied the energy transfer between CdSe/ZnS QDs with long ligands and graphene in a functionalized field-effect transistor structure, demonstrating that transfer processes for such a system can be indeed described by a Förster-like transfer model. We were able to explain the radiative-lifetime modification by a change in the Förster transfer rate between cQDs and the graphene channel of such FET structures. It can be concluded that by applying a back-gate voltage, we can alter the absorption of graphene with respect to the energy levels corresponding to photo-luminescence, i.e. radiative decay, of the cQDs. This gating principle indeed allows for an optimization of the efficiency of transfer to the graphene in such a FET device designed for photo-detection.

## Methods

### Sample Preparation

Several monolayer graphene samples of high quality grown by chemical vapour deposition (CVD) on silicon, similar to Kim *et al*.^1^, have been purchased from HEFEIVIGON TECHNOLOGY CO., LTD. The graphene was effectively isolated from the gate by a 280 nm silicon oxide layer grown on Si. A Nd:YAG laser and two PI motorized stages were employed to build a laser ablation setup^29^. Using this laser ablation setup, the graphene sample has been cut into 18 rectangles, with two of them pairwise connected by a graphene gate of approximately 140 μm by 100 μm size. Furthermore, a shadow mask has been fabricated from a 10 μm thick sheet of aluminium foil. This shadow mask has been used to deposit a 20 nm film of titanium following a 200 nm thick gold film as electric contact (cf. Fig. 1b). CdSe/ZnS cQDs with oleic acid (OA) as surface ligands were purchased from the Chinese company NajingTec, which were realized by a successive ion layer adsorption and reaction (SILAR) technique and finally dissolved in toluene. An average size of (5.5 ± 1.1) nm with uniform shape and normal size distribution was obtained from a statistical analysis of a transmission electron microscopy (TEM) image for these cQDs. The experimentally obtained quantum yield of the cQDs amounts to 60–70%, which was measured with an absorption spectroscopy setup, and compares well with the value of approximately 60% provided by the supplier. Photoluminescence, absorbance and emission lifetime of the cQDs are provided in the Supporting Information (cf. Fig. SI1). From a 5.26 μmolar solution, 2 μl were drop-casted on the sample and the solvent was evaporated (cf. Fig. SI2a). An analysis (cf. Fig. SI2b) shows that the film is thinner and more homogenous at its borders. A cross-sectional scanning electron microscopy (SEM) picture of a cQD-graphene FET sample shows a multilayer cQD coverage of graphene (cf. Fig. SI4). The ligand type was chosen to not bind covalently (the end group is a methyl group) in order to limit the modification of graphene’s electrical properties.

### Experimental Setup

The time-resolved photoluminescence (PL) measurements were performed using a frequency-doubled Nd:YAG laser (Quantel Brilliant) at 532 nm and 10 Hz repetition rate with 1.5 μJ per Pulse. The optical signal was collected and focused onto a spectrometer (Oriel Instruments MS-257) with a blaze grating of 300/500. Using a gated intensified charge coupled device (ICCD), transients of PL were measured. For current and bias voltage control, respectively, two current meters (Keithley 617) were employed. One of them was used to apply a 50 mV source-drain bias voltage to monitor the resistance of the graphene channel, while the other device was used to set the back-gate voltage. The gold contact pads on the sample were electrically contacted by tungsten needles (cf. Fig. 1). For the pump-intensity dependent measurements, a white-light continuous-wave (CW) source was used. For each pump intensity, both current meters were used to measure a resistance-response curve. The spectrum of the white-light lamp was used to identify the amount of light with wavelengths lower than 560 nm. This fraction of light corresponds to the spectral components absorbed by the QDs, thus, specified intensities correspond to that fraction.

## Additional Information

**How to cite this article**: Li, R. *et al*. Gate Tuning of Förster Resonance Energy Transfer in a Graphene - Quantum Dot FET Photo-Detector. *Sci. Rep.* **6**, 28224; doi: 10.1038/srep28224 (2016).

## Supplementary Material

Supplementary Information
