# Supplementary material for: Gate Tuning of Förster Resonance Energy Transfer in a Graphene - Quantum Dot FET Photo-Detector
Source: Sci Rep. 2016 Jun 20;6:28224. doi: 10.1038/srep28224 (PMC4913307; doi:10.1038/srep28224)
Supplement: Supplementary Information [file srep28224-s1.pdf]

Gate Tuning of Förster Resonance Energy Transfer in a Graphene - Quantum Dot FET Photo-Detector

Ruifeng Li[1,*,+]**, Lorenz Maximilian Schneider**[2,*,+]**, Wolfram Heimbrodt**[2]**,**
Huizhen Wu[1]**, Martin Koch**[2]**, and Arash Rahimi-Iman**[2]

[1]Department of Physics and the State Key Laboratory of Silicon Materials, Zhejiang University, Hangzhou, 310027,P.R. China

[2]Faculty of Physics and Materials Sciences Center, Philipps-Universität Marburg, 35032 Marburg, Germany

[+]these authors contributed equally to this work

[*]Address correspondence to: maximilian.schneider@physik.uni-marburg.de, lbrookcn@hotmail.com

Supporting Information

Characterization of the colloidal quantum dots (cQDs)

In the following, the optical characterization of the deployed CdSe/ZnS cQDs is briefly summarized. Firstly, continuous wave (CW) absorbance and photo luminescence measurements have been performed for the cQDs, which were dissolved in Toluene.(see Fig. SI.1a). Besides, time-resolved photo-luminescence data have been acquired in order to identify the intrinsic lifetime of the cQDs in solution (cf. Fig SI.1b).

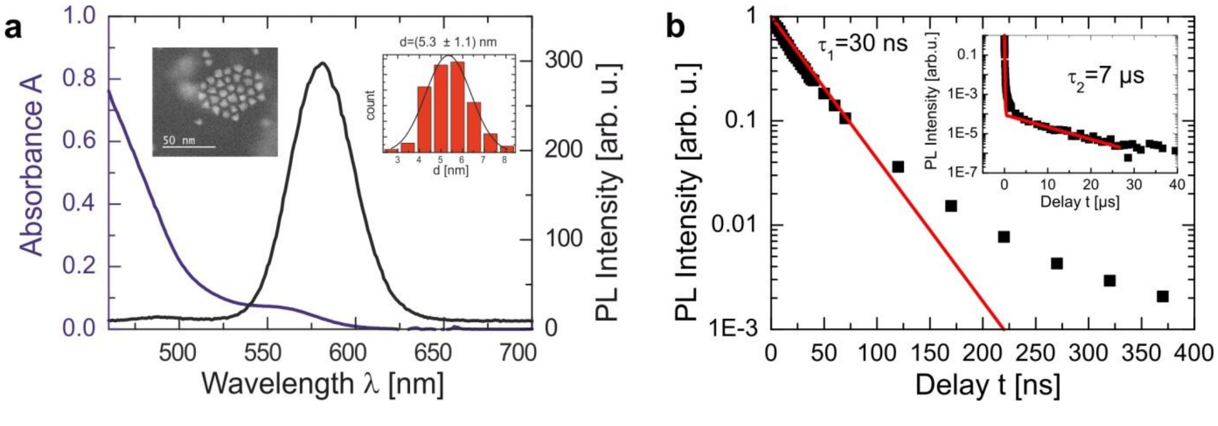

Figure SI.1 | Photoluminescence and absorbance of cQDs. a) Absorbance (red curve, right axis) and photoluminescence (black curve, left axis) of CdSe/ZnS QDs, respectively. Inset: Histogram of the size distribution of the used cQDs. Here, a representative TEM picture is shown, from which the QD sizes are retrieved. b) Decay of the QDs' photo-luminescence, represented by a logarithmically plotted transient, which has been fitted by a biexponential function. These cQDs have been subject to the studies on the hybrid cQD-graphene system. The inset presents a zoom-out overview on the recorded radiative decay up to 40 μs.

Drop Casting of cQDs onto a graphene transistor channel

Starting from a CAD model, a laser ablation setup was employed to cut the graphene layer on the substrate. The same technique was used to process a shadow mask in aluminium foil for the deposition of electrical contacts. Following the structuring and processing steps, several field-effect transistor (FET) structures were identified under an optical microscope. Functionalizing of the graphene-transistors was achieved by drop-casting of the aforementioned cQD solution onto the channel region of those FET structures. For individually prepared samples, a representative camera picture is shown which was recorded under UV illumination (see Fig. SI.2a). To estimate the homogeneity of the QD layer, the brightness of signal in the recorded picture was analysed along the channel axis. A representative cross-section of brightness is presented in Fig. SI.2b, with labelled arrows and dashed-lines marking key locations on the sample in the graph. In this picture, two FET structures are covered, with the UV light exciting an expanded area of cQDs (cf. Fig. SI.2a). The dashed lines in the brightness chart represent the contact pads. Naturally, areas with higher brighness than their surroundings correspond to regions with metallic surfaces underneath the cQD layer. A pronounced dip in the center of each highlighted contact-pad region represents the corresponding gate region in this chart. Indeed, the highest cQD concentration is found on the FET structure in the center of the recorded picture.

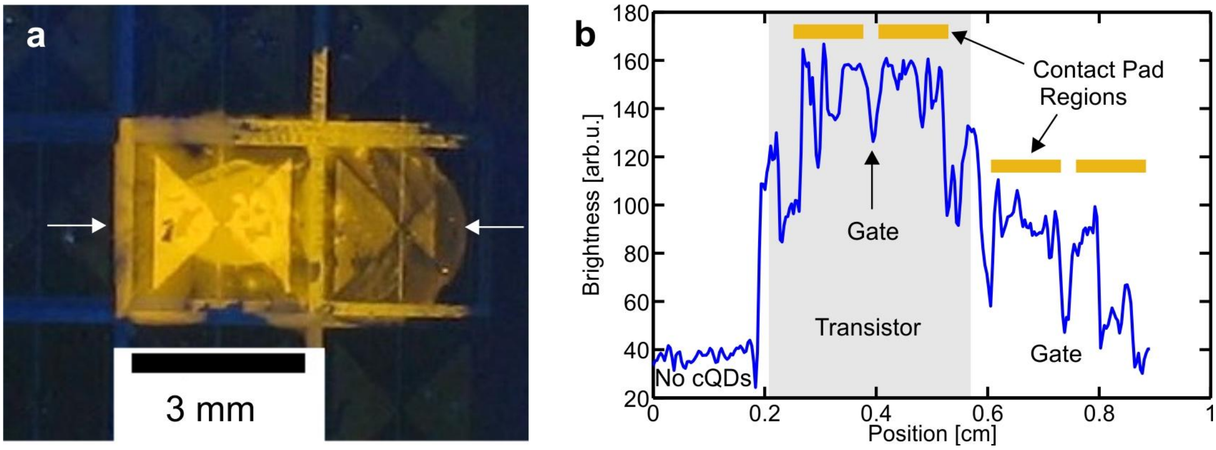

Figure SI.2 | cQD-covered field-effect transistor structure. a) Representative camera picture of drop-casted cQDs on agraphene FET. The white arrows indicate the cross-section at which a brightness analysis was performed. b) The analysis of brightness along the channel axis clearly shows contact-pad regions with a dip in the center, which allows one to distinguish between the gate of a structure and the surrounding contact pads. This is explained by the stronger quenching of signal by the graphene areas compared to the gold-covered regions. Furthermore, the analysis shows that in the center region, the cQD drop is rather inhomogenous, thus showing strong brightness variation. On the second FET that is neighboring the centrally displayed FET structure, a more homogenous film is obtained.

Electrical contacts

The gold contact pads on the sample were electrically contacted by tungsten needles. Thus, one can expect a contact resistance to affect the channel resistance measurements. Assuming that all pieces of graphene on the sample, i.e. the graphene channels in every structure, exhibit the same resistance, we estimate contact resistances based on the lower limit of the measured source-drain resistance, which can be different in magnitude due to a varying quality of the established electrical contact for each structure. Here, we did not distinguish contact

resistances for source and drain contacts and estimate $R_{Contact}$ for the 1st, 2nd and 3rd transistor to be about 0-350, 0-5, and 450-460 Ohms, respectively, with the spread of those attributed to different contact qualities.

Transients of the cQD PL measurements on the graphene FET

The transients of the cQD PL have been acquired as described in the method section and fitted by mono exponential fits. For all three cQD-graphen photodetecting structures studied, transients were recorded as a function of the gate voltage, respectively. The plots shown in Fig. SI.3 include monoexponential fits to the transients used to extract the radiative lifetime.

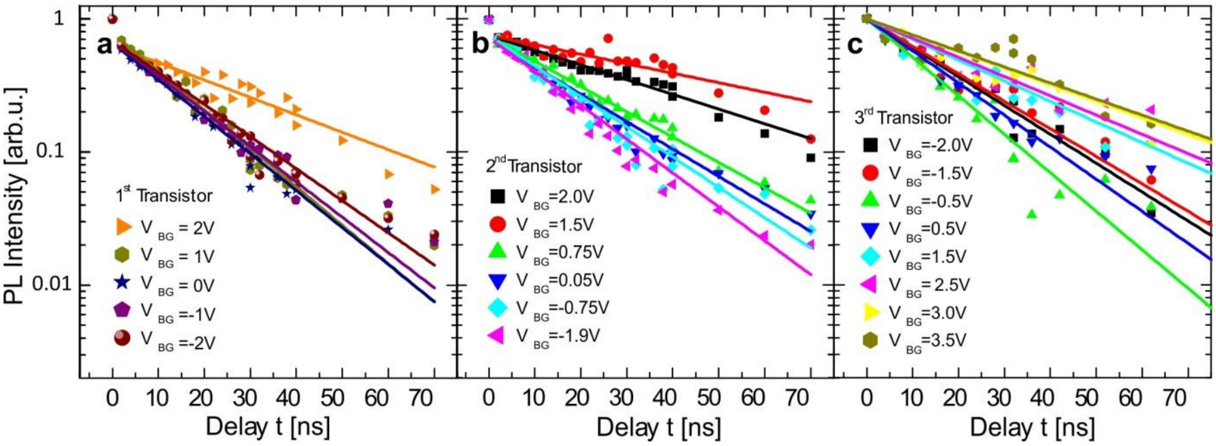

Figure SI.3 | Transient photoluminescence of cQDs on gated graphene. Transients obtained from the 1st (a), 2nd (b) and 3rd (c) cQD-graphen FET structure, respectively. The plots include monoexponential fits to the transients used to extract the radiative lifetime.

Structure analysis of cQDs-covered graphene FET

Figure SI.4 (a) shows the cross-section image of a cQD-covered FET structure that was obtained from scanning electron microscopy (SEM). Limited by the resolution of SEM, the monolayer of graphene is not observable. A 100 nm thick layer of cQDs resulted from drop-casting and provided closely packed cQDs in a multilayer configuration on the graphene FET structure, approximately equivalent to approximately 20 layers of cQDs. Monolayer graphene is revealed in Raman spectra, showing a sharp characteristic peak at 2700 cm^{-1} [SI-1].

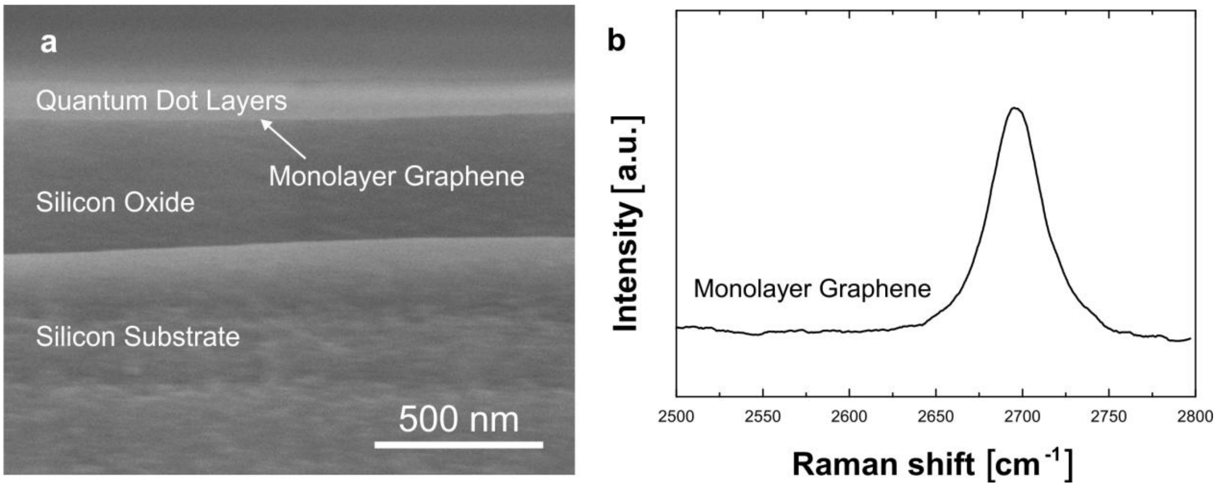

Figure SI.4 | Structure analysis of cQDs-covered graphene FET. a) SEM image of a cQD-covered FET structure. b) Raman spectrum of monolayer graphene.

Three different transistor devices based on the same fabrication technique

Figure SI.5 shows microscopy images of three different graphene FET structures, which were based on the same design and fabricated accordingly by the same technique. A similar coverage of graphene with cQDs was achieved by carefully drop-casting the same amount of solution on each FET structure, which is reflected by the intensity of photoluminensce in time-resolved spectroscopy measurements. Owing to fabrication tolerances, minor differences in the performance of the cQD-graphene photo-detectors can be observed in the experiment.

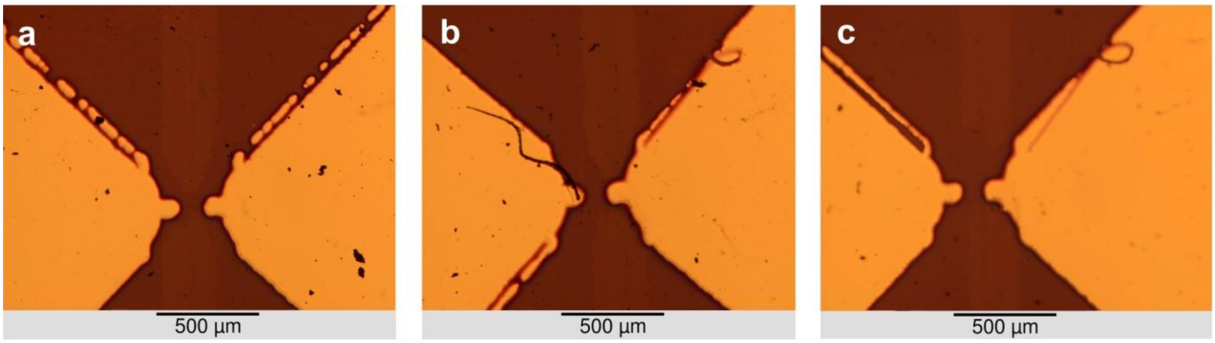

Figure SI.5 | Microscopy images of three different graphene FET structures. All structures were based on the same design and fabricated accordingly by the same technique.

Time-integrated spectra

Figure SI.6 demonstrates that in our experiment no spectral peak shift as a function of the back-gate voltage is observed.

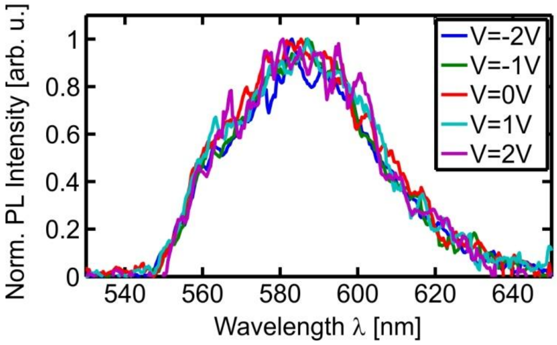

Figure SI.6 | Time-integrated photoluminescence spectra. Here, the data is integrated over the first 72 ns of the recorded transient PL. No spectral peak shifts are observed in the presence of different back-gate voltages**.**

Reference

[SI-1] Ferrari A C, Basko D M. Raman spectroscopy as a versatile tool for studying the properties of graphene. Nature Nanotechnology **8(4)**, 235-246 (2013)
